# Drug-related problems and associated factors among patients with kidney dysfunction at a tertiary hospital in southwestern Uganda: a prospective observational study

**DOI:** 10.1186/s12882-023-03437-2

**Published:** 2023-12-19

**Authors:** Julius Kyomya, Fredrick Atwiine, Efrata Ashuro Shegena, Rose Muhindo, Tadele Mekuriya Yadesa

**Affiliations:** 1 Department of Pharmacy, Faculty of Medicine, Mbarara University of Science and Technology, P. O. Box 1410, Mbarara, Uganda; 2 Department of Internal Medicine, Faculty of Medicine, Mbarara University of Science and Technology, P. O. Box 1410, Mbarara, Uganda; 3 Department of Clinical Pharmacy and Pharmacy Practice, School of Pharmacy, Kampala International University, Ishaka, Uganda

**Keywords:** Drug-related problem, Kidney dysfunction, Acute kidney injury, Chronic kidney disease, Medication management

## Abstract

**Background:**

Kidney dysfunction is a common, progressive condition that is increasingly becoming a global public health issue. Because the kidneys are the major route for drug excretion, impaired renal function can change the pharmacokinetics and pharmacodynamics of drugs that are renally excreted. Additionally, patients with kidney dysfunction often have co-morbidities and the associated use of multiple medications which increases the risk of drug-related problem (DRP) occurrence. This study aimed to determine the prevalence, types, and factors associated with DRPs in patients with kidney dysfunction.

**Method:**

We conducted a prospective observational study over 3 months among hospitalized patients diagnosed with acute kidney injury or chronic kidney disease who were hospitalized in the medical ward, and patients attending the renal outpatient clinic at Mbarara Regional Referral Hospital. A total of 183 participants were enrolled through the use of a consecutive sampling technique. DRPs were classified according to the PCNE classification version 9.1. Data analysis was carried out using SPSS version 25.

**Results:**

A total of 174 patients with kidney dysfunction were included in the study with a mean ± SD age of 50.34 ± 18.13 years. A total of 219 DRPs were incurred by 138 (79.3%) study participants. The most common DRPs were ‘Untreated symptoms or indication’ (35.6%) followed by ‘adverse event (possibly) occurring’ (28.3%), and ‘effect of drug treatment not optimal’ (23.3%). Antimicrobials were the most involved drugs in suboptimal drug treatment (31.3%) and unnecessary drug treatment (32.1%). The study showed that length of hospital stay ≥ 5 days (AOR = 6.39, 95% CI: 1.75–23.27; *p*-value = 0.005) significantly increased the risk of DRP occurrence.

**Conclusion:**

The current results, in agreement with previous literature, showed a high burden of DRPs among patients with kidney dysfunction. Antimicrobials were the most involved drugs in suboptimal as well as in unnecessary drug treatment. Longer hospital stay significantly increased the risk of DRPs. The high prevalence of DRPs in patients with kidney dysfunction and the potential impact on antimicrobial resistance underscores the importance of regular medication reviews and close monitoring of patients with renal dysfunction.

## Introduction

Kidney dysfunction is the inability of the kidneys to adequately filter toxins and waste products from the blood due to a reduction in the glomerular filtration rate. Functional abnormalities of the kidneys can be detected through various measures such as estimated glomerular filtration rate(eGFR), proteinuria/albuminuria, and urine output [1]. Kidney dysfunction is a common, progressive condition that is increasingly becoming a global public health issue [2, 3]. Kidney disease is the 10th leading cause of death worldwide accounting for 1.3 million deaths annually [4].

Over the last 30 years, the contribution of non-communicable diseases, particularly kidney dysfunction, to morbidity and mortality in Sub-Saharan Africa has increased [5]. A 2018 meta-analysis of 98,432 participants from 98 studies in Africa found a 15.8% overall prevalence of chronic kidney disease stages 1–5 [6], while a cross-sectional pilot study in Uganda found a high prevalence of proteinuria and a lower eGFR among urban Kampala residents, indicating some degree of kidney impairment [7].

Kidney dysfunction may present in several forms including acute kidney injury (AKI) or chronic kidney disease (CKD) both of which are significant global health challenges [8, 9]. Impaired kidney function can change the pharmacokinetics and pharmacodynamics of a drug that is largely removed by renal excretory systems. This results in the accumulation of toxic levels of the drug or its metabolites [10]. Comorbid conditions are also common in patients with kidney dysfunction, either as a cause or as a result of the dysfunction. Additionally, the management of kidney dysfunction is mostly based on long-term drug therapy to prevent disease progression, morbidity, and mortality [11]. This places patients with kidney dysfunction at a higher risk of encountering drug-related problems (DRPs) [12, 13].

According to Pharmaceutical Care Network Europe (PCNE), a DRP is “*an event or circumstance involving drug therapy that actually or potentially interferes with desired health outcomes*” [14]. DRPs have become common safety issues in hospitalized patients causing harm to the patient and increased healthcare costs in recent years [15, 16]. Kidney dysfunction is a significant risk factor for DRP occurrence not just because of the disease and its associated comorbidities, but also because of the medication use burden in these patients [11]. If not addressed DRPs may lead to poor health outcomes and disease progression in patients with kidney dysfunction [17].

Early detection and prevention of DRPs among patients with kidney dysfunction in developed countries has been made possible by the incorporation of clinical pharmacy services and the application of evidence-based guidelines [18, 19]. This has led to a comprehensive and interdisciplinary approach to identify, prevent and manage DRPs in these patients [19, 20]. In particular, pharmacist-led interventions have successfully identified and resolved DRPs in patients with kidney dysfunction [21]. The reduction of DRPs in patients with kidney dysfunction may improve the quality of life and reduce morbidity, mortality, and associated healthcare costs [22].

Although DRPs have been well-studied in patients with kidney dysfunction in high-income countries, there is a dearth of literature on the specific risk factors and impact of these problems in Sub-Saharan Africa. A few studies from these settings have identified varying prevalence rates of DRPs among CKD patients; 100% in Kenya [23], 78.6% in Ethiopia [24], and 70.03% in Nigeria [12]. These variations could be explained by differences in patient characteristics and healthcare practices.

This study aimed to assess the prevalence, types, and risk factors associated with DRPs in patients with kidney dysfunction at Mbarara Regional Referral Hospital (MRRH), using the PCNE classification tool.

## Methods

### Study design and setting

A prospective cross-sectional study was conducted among hospitalized patients with a confirmed diagnosis of AKI or CKD with eGFR of less than 60 mL/min/1.73m^2^ at the MRRH medical ward and renal clinic from October 2022 to January 2023. MRRH is a tertiary hospital and the largest referral center in southwestern Uganda, 280 km from the capital Kampala with a 350-bed capacity. The hospital serves a population of over four million people in its catchment area comprising 13 districts of southwestern Uganda. The medical in-patient ward has 50 beds and 300 patients are expected to be admitted each month. The hospital has a renal clinic which opened in 2011 and is part of the various units under the internal medicine department. There are currently two nephrologists and one nurse operating the clinic. The clinic offers outpatient services for patients with kidney diseases and conducts its operations once a week on a Monday. There is also a dialysis unit with seven dialysis machines that offer hemodialysis services to patients with end-stage renal disease (ESRD) and acute kidney injury.

### Study participants

We included all patients of both genders aged 18 years and above admitted to the medical ward or attending the renal clinic of MRRH during the study period, with a confirmed diagnosis of AKI or CKD with eGFR of less than 60 mL/min/1.73m^2^, and willing to participate in the study. The eGFR was calculated using the CKD-EPI Creatinine Eq. (2021) [25]. Participants who were critically sick and unable to take an effective interview were excluded from this study.

### Sample size

The sample size was calculated using Fisher’s formula [26] based on the estimated prevalence of 87% [16] and the assumptions of a 95% confidence interval, and a 5% margin of error. Thus, a sample size of 174 was required. By adding 5% non-response rate, the final computed sample size was 183 participants. A consecutive sampling technique was used during the study period and data collection continued until the sample size was achieved.

### Data collection tools and procedures

A structured data collection tool was created based on carefully analyzed published articles in the literature. Part 1 of the tool is a questionnaire that was used to collect the patient’s sociodemographic and clinical characteristics, while part 2 is a data abstraction form that was used to collect disease, laboratory investigations, and drug-related data from the patient’s chart. The identified DRPs were then categorized using PCNE classification version 9.1 [14]. Discussions with treating physicians and residents provided further information and explanations on some patients’ medical information. Patients’ charts were also reviewed to obtain relevant disease and drug-related data. The clinical pharmacist through a review of the collected data and patient interaction was then able to evaluate for possible DRPs. The PCNE classification system version 9.1 was used primarily to categorize DRPs by problem type. While we also attempted to identify and categorize the causes of these problems where possible, our main focus was on the identification and categorization of the problems. The clinical pharmacists’ evaluations and recommendations were based on KDIGO guidelines for the management of AKI, CKD, comorbidities in AKI & CKD, and complications of AKI & CKD [27].

### Data analysis

Data analysis was carried out using Statistical Package for Social Sciences (SPSS), version 25 (SPSS Inc., Cary, NC, USA). The sociodemographic and clinical characteristics of individuals were presented using descriptive statistics such as mean and percentages. The prevalence and types of DRPs are reported in percentages. We used binary logistic regression to determine the association between independent and dependent variables. Differences between variables with a *p*-value < 0.25 were adopted for multivariate analysis, and differences with a *p*-value < 0.05 were considered statistically significant.

## Results

### Participant enrolment

The study involved 183 adult participants and data from 9 of them was excluded due to incomplete or duplicate information. The final analysis was conducted on data from 174 participants.

### Participants’ sociodemographic, clinical, and drug characteristics

The majority of the participants in this study were male 109 (62.6%) aged between 18 and 90 years with a mean ± SD of 50.3 ± 18.1 years. Only 27 (15.5%) participants reported a history of smoking while 79 (45.4%) reported an alcohol consumption history. About 112 (64.4%) participants were receiving five or more drugs and the majority of the participants 168 (96.6%) reported having received counseling on the use of their medication (Table 1).

**Table 1:** Sociodemographic and clinical characteristics of patients with kidney dysfunction at MRRH

Characteristic N = 174	Value
**Male gender**, n (%)	109 (62.6)
**Age**, mean ± SD -years-	50.3 ± 18.1
**Level of education**, n (%)	
None Primary school Secondary school College or University level	28 (16.1) 83 (47.7) 41 (23.6) 22 (12.6)
**Current employment status**, n (%)	
Worker Employee Self-employed	138 (79.3) 26 (14.9) 10 (5.7)
**Smoking history**, n (%)	27 (15.5)
**History of alcohol use**, n (%)	79 (45.4)
**Type of kidney disease**, n (%)	
Acute Kidney Injury Chronic Kidney Disease	92 (52.9) 82 (47.1)
^**a**^**Polypharmacy**, n (%)	112 (64.4)
**Counselled on the use of their medication**, n (%)	168 (96.6)
**Number of comorbidities, n (%)**	
None One Two or more	35 (20.1) 96 (55.2) 43 (24.7)
**Hypertension**, n (%)	81 (46.6)
**Diabetes**, n (%)	30 (17.2)
**HIV**, n (%)	34 (19.5)
**Heart Failure**, n (%)	18 (10.3)
**Length of hospital stay**, median (IQR) -days-	4 (2–6)
**eGFR**, median (IQR) -ml/min/m^3^-	25 (9–45)

^*a*^ *Five or more drugs; SD = standard deviation, IQR = Interquartile range*

**Fig. 1:**
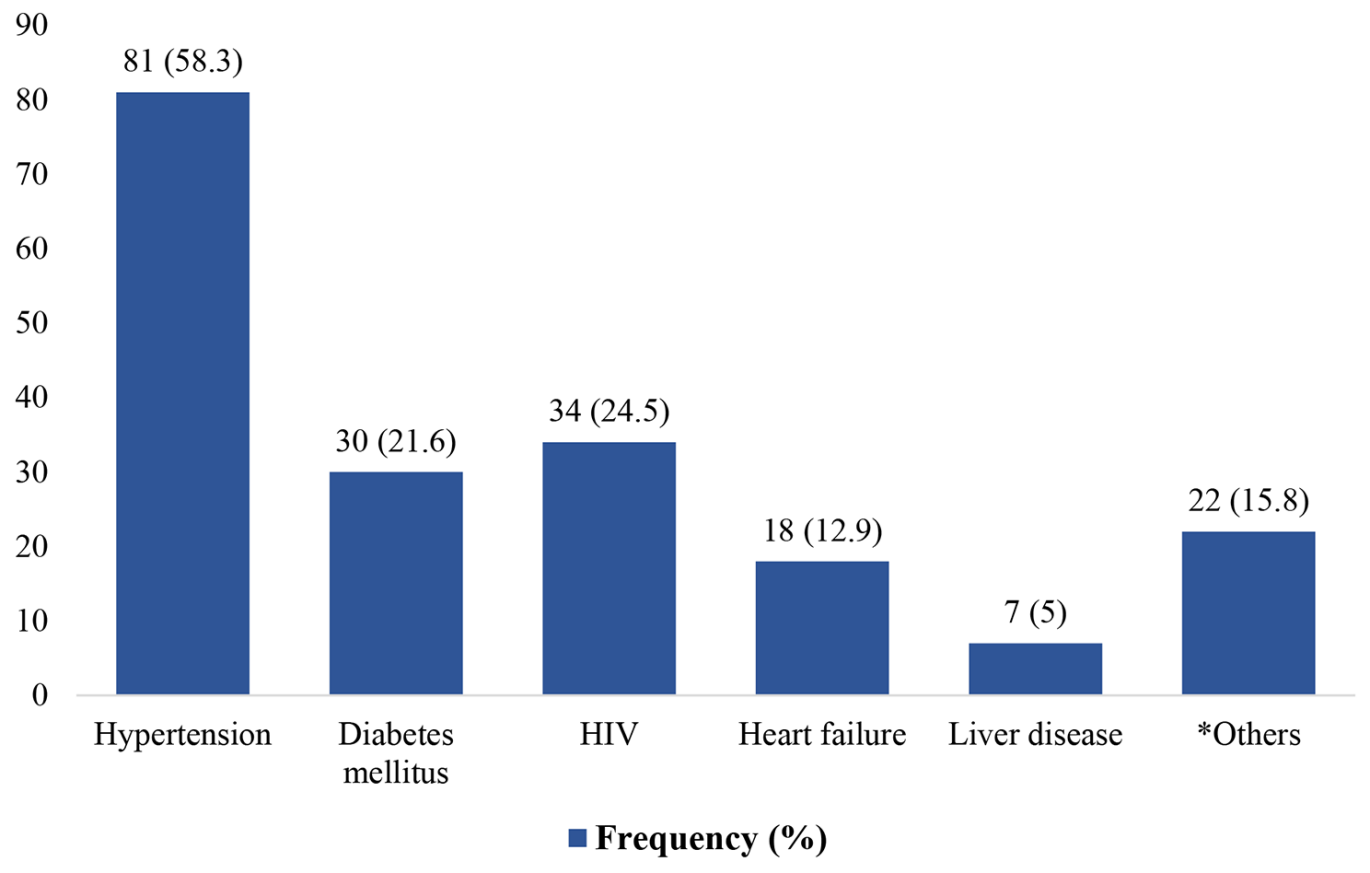
Common comorbid conditions among patients with kidney dysfunction receiving care at MRRH. HIV = Human Immunodeficiency Virus *COPD, Malaria, Asthma, Pulmonary TB, Epilepsy, Hepatitis C, DVT, Brucellosis, medullary aplasia, Urinary Tract Infection, Esophagitis

**Fig. 2:**
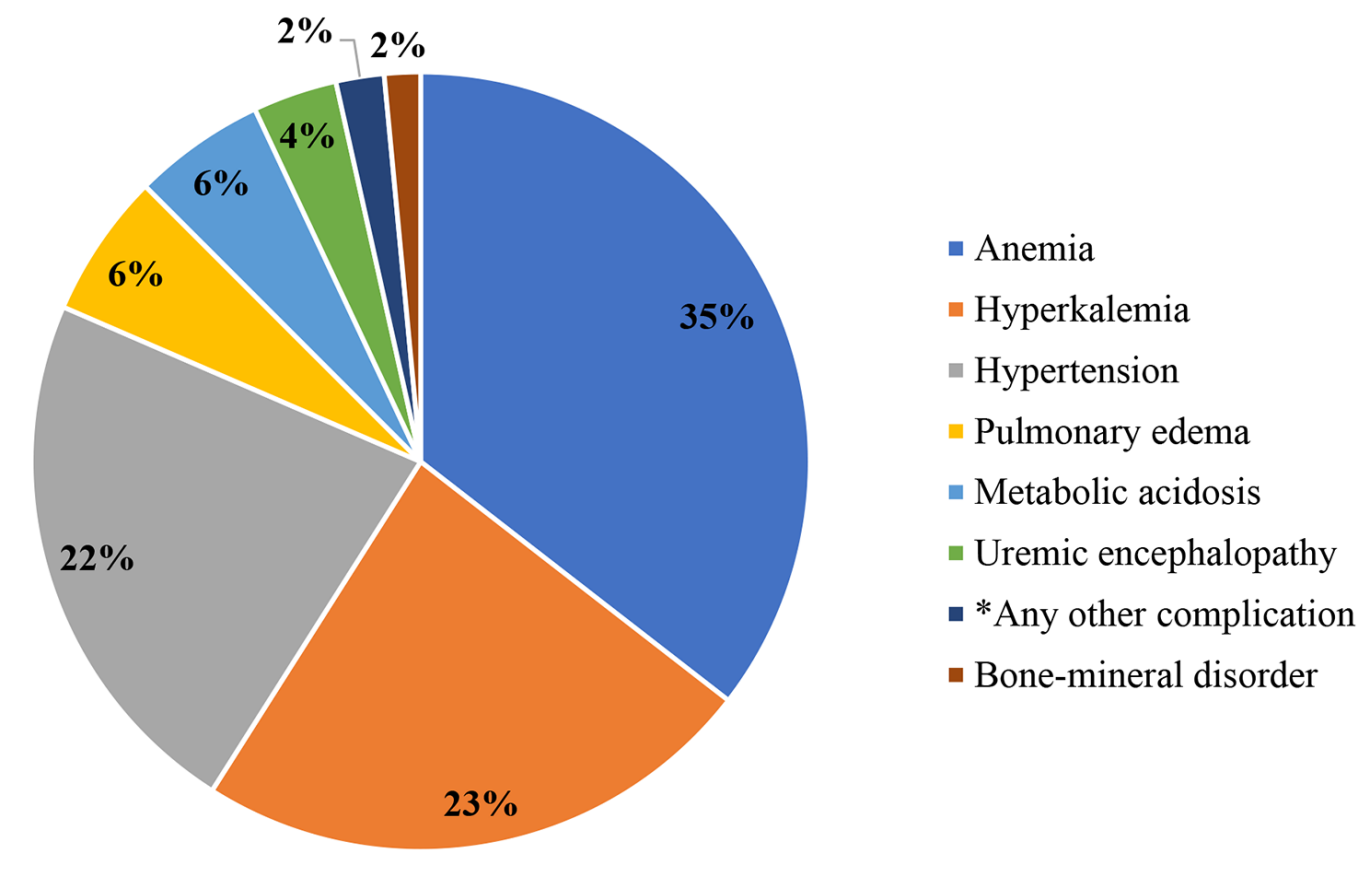
Complications of kidney disease among patients with kidney dysfunction at MRRH. *Hypokalemia (2), Uremia (1), and Uremic gastropathy (1)

The most common comorbidity among the study participants was hypertension (46.6%) (Fig. 1) 19.5% had HIV, and 17.2% had diabetes mellitus (Table 1). The distribution of these chronic comorbidities also varied between patients with AKI and those with CKD as shown in Table 2. Anaemia 71 (35%) was the most common complication of kidney disease among the study participants followed by hyperkalemia 47 (23%) and hypertension 45 (22%) (Fig. 2).

**Table 2:** Distribution of comorbidities across AKI and CKD

	Type of Kidney disease		Total
	Acute kidney injury Frequency (%)	Chronic kidney disease Frequency (%)	
Hypertension	29 (43.9%)	52 (71.2%)	81
Diabetes mellitus	10 (15.2%)	20 (27.4%)	30
HIV	19 (28.8%)	15 (20.5%)	34
Heart failure	7 (10.6%)	11 (15.1%)	18
Liver disease	6 (9.1%)	1 (1.4%)	7
Others*	12 (18.2%)	10 (13.7%)	22

* *COPD, Malaria, Asthma, Pulmonary TB, Epilepsy, Hepatitis C, DVT, Brucellosis, medullary aplasia, Urinary Tract Infection, Esophagitis*

### Prevalence of drug-related problems

**Fig. 3:**
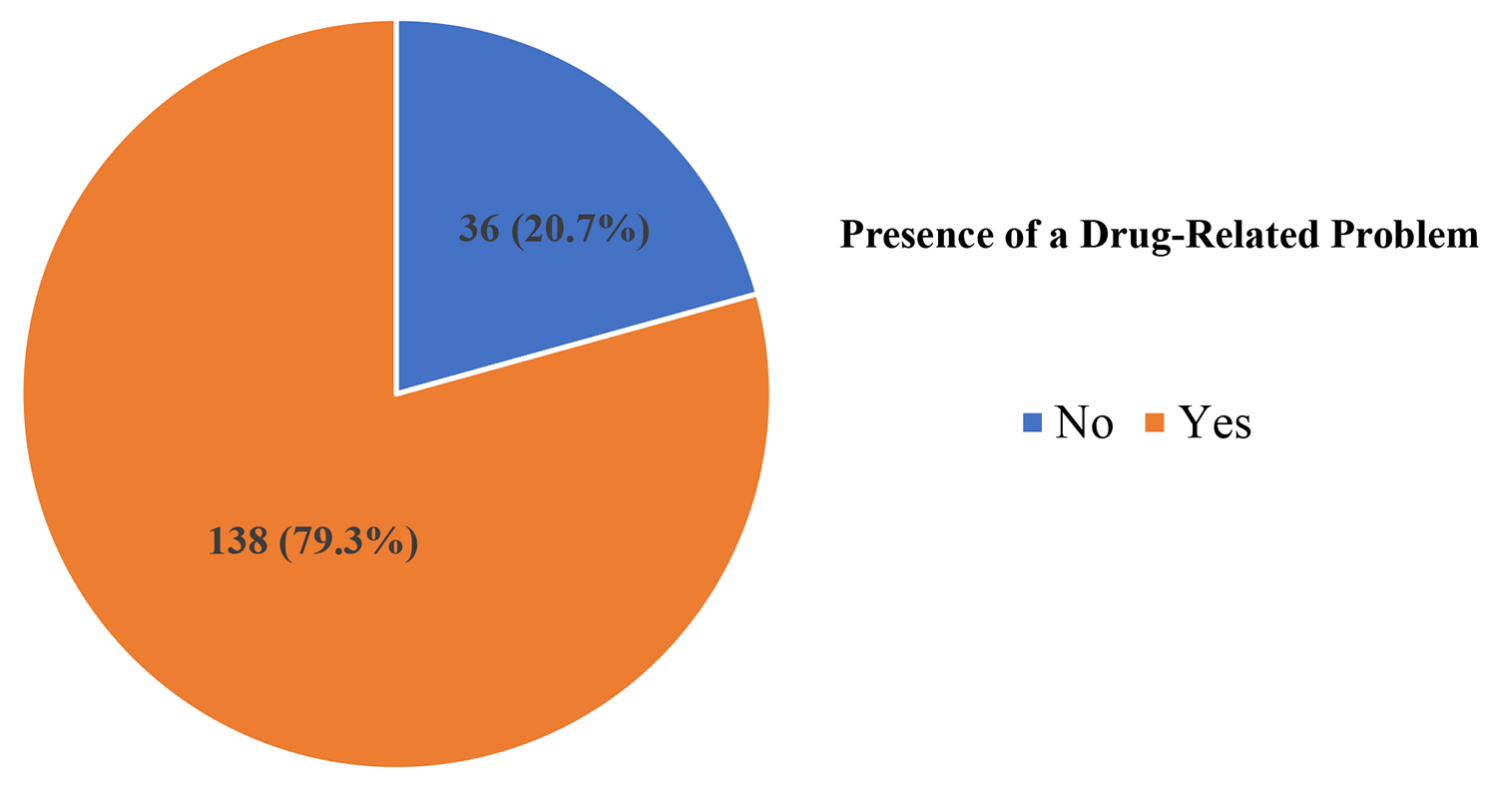
Prevalence of drug-related problems among patients with kidney dysfunction at MRRH

Out of 174 patients included, 138 participants had at least one drug-related problem making an overall prevalence of 79.3% (Fig. 3*)*. The majority of the participants 80 (46.0%) had one DRP, 39 (22.4%) had two DRPs, and 19 (10.9%) had three or more DRPs.

### Types of drug-related problems among patients with kidney dysfunction at MRRH

**Fig. 4:**
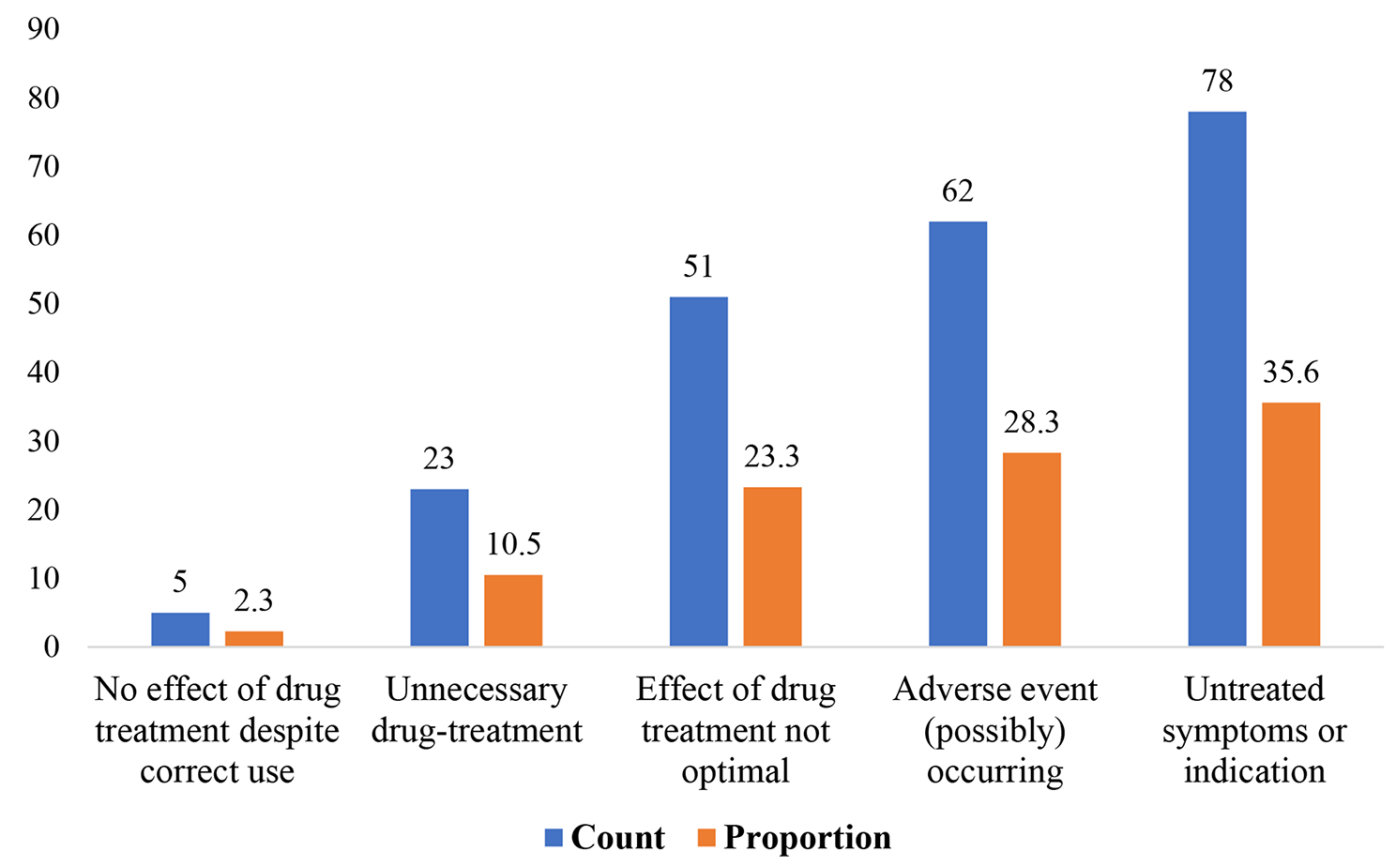
Types of drug-related problems identified among patients with kidney dysfunction at MRRH

A total of 219 DRPs were incurred by 138 patients; a mean of 1.26 DRPs per patient. ‘Untreated symptoms or indication’ was the most common DRP at 35.6%, followed by ‘adverse event (possibly) occurring’ at 28.3%, and ‘effect of drug treatment not optimal’ at 23.3% (Fig. 4). Several causes were identified for each type of DRP and presented in Table 3.

**Table 3:** Causes of the different DRPs identified among patients with kidney dysfunction at MRRH

Drug-related problem	Cause of the drug-related problem	Frequency
‘No effect of drug treatment despite correct use’	Inappropriate combination of drugs, drugs and herbal medications, or drugs and dietary supplements	1
	Inappropriate timing or dosing intervals	1
	Medication reconciliation problem	1
‘Effect of drug treatment not optimal’	Incomplete drug treatment despite existing indication	15
	Drug dose too low	13
	The dosage regimen is not frequent enough	8
	Short treatment duration	6
	Inappropriate drug form/formulation (for this patient)	4
	Drug not administered at all by a health professional	2
	Inappropriate timing or dosing intervals	2
	Patient intentionally uses/takes less drug than prescribed or does not take the drug at all for whatever reason	2
	Necessary information not provided	1
	Prescribed drug not available	1
‘Untreated symptoms or indication’	No or incomplete drug treatment in spite of existing indication	63
	Medication reconciliation problem	5
‘Adverse event (possibly) occurring’	Inappropriate drug according to guidelines/formulary	30
	Dosage regimen too frequent	14
	Drug dose of a single active ingredient too high	10
	Inappropriate combination of drugs	2
	Too many different drugs/active ingredients prescribed for an indication	2
	Drug dose too low	1
	Medication reconciliation problem	1
	The patient decides to use unnecessary drug	1
‘Unnecessary drug treatment’	No indication for drug	15
	Inappropriate drug form/formulation (for this patient)	4
	Inappropriate duplication of therapeutic group or active ingredient	3
	Medication reconciliation problem	2
	Too many different drugs/active ingredients prescribed for indication	2

### Medications used in the management of the study participants

**Fig. 5:**
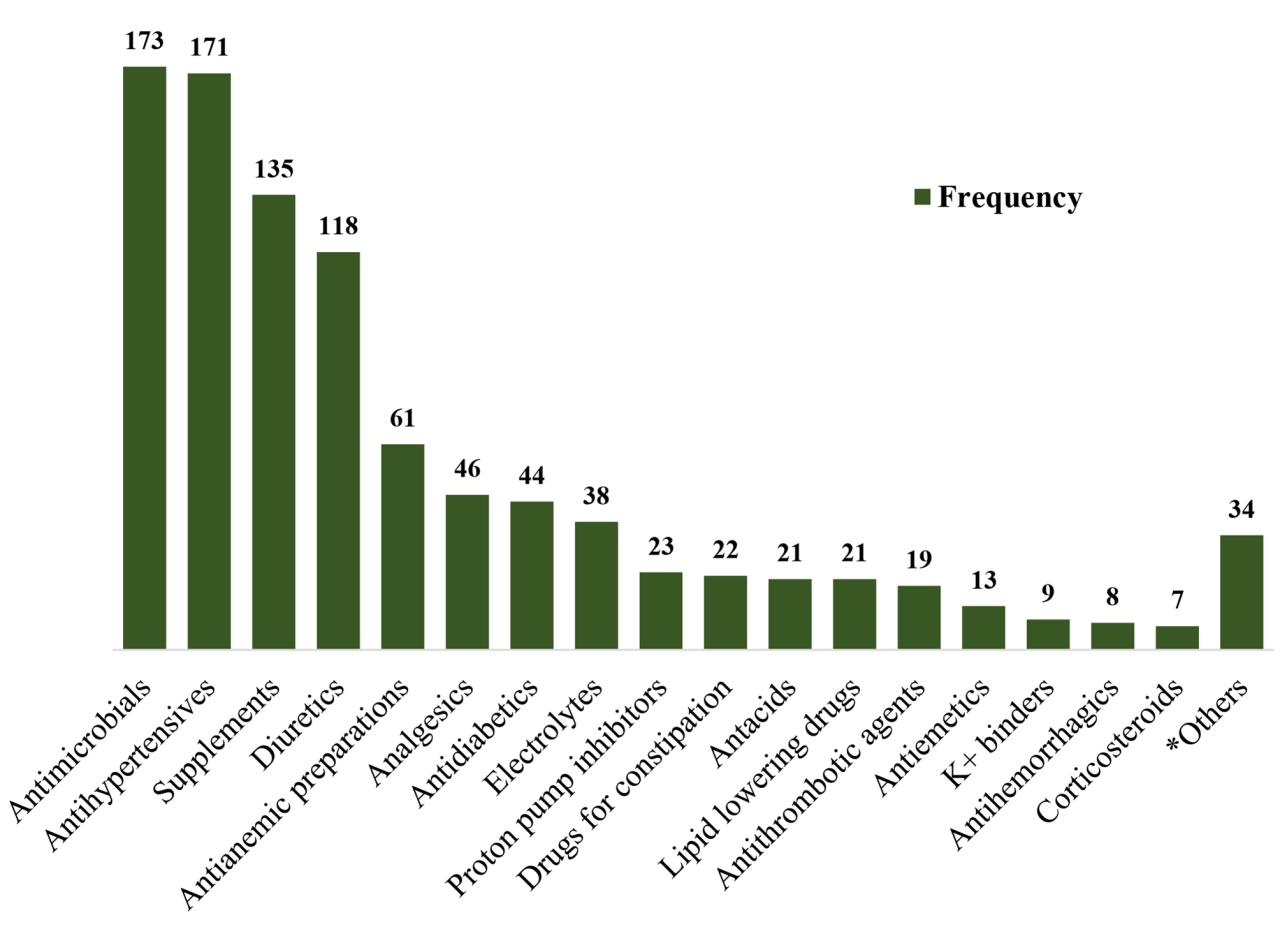
Prescribed drugs among patients with kidney dysfunction at MRRH. *Adrenergics (5), Immunosuppressants (4), Antiepileptics (3), Antipsychotics (3) Cardiac glycosides (3), Cough and cold preparations (3), Antidepressants (2), Antihistamines (2), Albumin (2), Cardiac stimulants (2), Hormonal preparations (2), Antidiarrheals (1), Antigout preparations (1), Muscle relaxants (1)

Drugs used by the study participants are summarized in Fig. 5. The median number of drugs prescribed was 5 with an interquartile range of 4 to 7 drugs. The most commonly prescribed drugs among the study participants were antimicrobials 173, antihypertensives 171, supplements 135, and diuretics 118 respectively. Antimicrobial agents were the most involved drugs in DRPs ‘effect of drug treatment, not optimal’, ‘adverse event (possibly) occurring’, and ‘Unnecessary drug treatment’ as shown in Table 4.

**Table 4:** Drugs involved with the different drug-related problems among patients with kidney dysfunction at MRRH

Drug-related problem	ATC classification of the drugs involved (Frequency)
‘No effect of drug treatment despite correct use’	Methyldopa (3), Calcium channel blockers (3), Beta blocking agents (2), Diuretics (1), Iron preparations (1)
‘Effect of drug treatment not optimal’	Antimicrobials (15), Calcium channel blockers (9), Diuretics (8), Iron preparations (3), Methyldopa (3), Drugs for constipation (3), Proton pump inhibitors (2), Lipid modifying agents (1), Beta blocking agents (1), Analgesics (1), Electrolytes (1), Antiepileptics (1)
‘Adverse event (possibly) occurring’	Antimicrobials (18), Agents acting on the RAAS (9), Drugs used in diabetes (8), Iron preparations (7), Analgesics (6), Diuretics (5), Beta blocking agents (4), Proton pump inhibitors (4), Antithrombotics (2), Calcium channel blockers (2), Cardiac glycosides (2), Antidepressants (1), Methyldopa (1), Hydralazine (1), Drugs for constipation (1), Pituitary and hypothalamic hormones (1)
‘Unnecessary drug treatment	Antimicrobials (9), Beta blocking agents (4), Lipid modifying agents (3), Adrenergics (2), Antacids (2), Corticosteroids (2), Electrolytes (2), Cardiac glycosides (1), Diuretics (1), Drugs used in constipation (1), Proton pump inhibitor (1).

### Factors associated with drug-related problems

The association of independent variables with drug-related problems was investigated using both univariate and multivariate logistic regression techniques. In univariate logistic regression analysis, employment status (COR = 3.01, 95% CI: 0.79–11.48; *p*-value = 0.106), alcohol history (COR = 1.99, 95% CI: 0.81–4.91; *p*-value = 0.211), patients unable to afford prescribed medicine (COR = 5.93, 95% CI: 0.77–45.78; *p*-value = 0.088), HIV comorbidity (COR = 1.83, 95% CI: 0.78–4.28; *p*-value = 0.166), and length of hospital stay greater than 5 days (COR = 4.97, 95% CI: 1.63–15.10; *p*-value = 0.005) all had a *p*-value < 0.25 and were introduced into the multiple logistic regression.

One variable retained statistical significance in the multivariate analysis which is the length of hospital stay greater > = 5 days (AOR = 6.39, 95% CI: 1.75–23.27; *p*-value = 0.005) compared to 2 or fewer days (Table 5).

**Table 5:** Univariate and multivariate logistic regression of the factors associated with DRPs among patients with kidney dysfunction at MRRH

Variables	Category	DRP		COR (95% CI)	*P* value	AOR (95% CI)	*P* value
		No	Yes				
Gender	Male	22 (20.2)	87 (79.8)	1.09 (0.51–2.31)	0.831		
	Female	14 (21.5)	51 (78.5)	1			
Age group	< 50	20	68	1			
	>=50	16	70	1.29 (0.62–2.69)	0.503		
Employment status *	Worker	25 (18.1)	113 (81.9)	3.01 (0.79–11.48)	**0.106**	4.43 (0.70-27.95)	0.113
	Employee	7 (26.9)	19 (73.1)	1.81 (0.39–8.39)	0.448	7.61 (0.85–68.14)	0.07
	Self-employed	4 (40)	6 (60)	1			
Smoking history	No	32 (21.8)	115 (78.2)	1			
	Yes	4 (14.8)	23 (85.2)	1.6 (0.52–4.96)	0.416		
Alcohol history *	No	23 (24.2)	72 (75.8)	1			
	Yes	13 (16.5)	66 (83.5)	1.62 (0.76–3.46)	**0.211**	1.99 (0.81–4.91)	0.136
Patients able to afford prescribed medicine*	No	1 (4.8)	20 (95.2)	5.93 (0.77–45.78)	**0.088**	2.0 (0.23–17.58)	0.534
	Yes	35 (22.9)	118 (77.1)	1			
Medicine use without a prescription	No	30 (20.5)	116 (79.5)	1.06 (0.39–2.83)	0.916		
	Yes	6 (21.4)	22 (78.6)	1			
Herbal medicine use	No	34 (21.5)	124 (78.5)	1			
	Yes	2 (12.5)	14 (87.5)	1.92 (0.42–8.86)	0.403		
Number of comorbidities	None	9 (25.0)	27 (75.0)	1			
	One	20 (21.1)	75 (78.9)	1.25 (0.51–3.08)	0.628		
	Two or more	7 (16.3)	36 (83.7)	1.71 (0.57–5.18)	0.34		
Type of kidney disease	AKI	19 (20.9)	72 (79.1)	0.747 (0.36–1.56)	0.436		
	CKD	17 (20.5)	66 (79.5)	1			
Polypharmacy	No	4 (21.1)	15 (78.9)	1			
	Yes	32 (20.6)	123 (79.4)	1.03 (0.32–3.30)	0.967		
Hypertension	No	22 (23.7)	71 (76.3)	1			
	Yes	14 (17.3)	67 (82.7)	1.48 (0.70–3.14)	0.302		
HIV*	No	26 (18.6)	114 (81.4)	1.83 (0.78–4.28)	**0.166**	1.6 (0.57–4.51)	0.386
	Yes	10 (29.4)	24 (70.6)	1			
Anaemia	No	8 (18.2)	36 (81.8)	1.33 (0.55–3.21)	0.527		
	Yes	26 (22.8)	88 (77.2)	1			
Length of hospital stay*	<=2 days	14 (31.1)	31 (68.9)	1			
	3–4 days	17 (24.6)	52 (75.4)	1.38 (0.60–3.19)	0.449	1.76 (0.65–4.77)	0.268
	>=5 days	5 (8.3)	55 (91.7)	4.97 (1.63–15.10)	**0.005**	6.39 (1.75–23.27)	**0.005**

* *Variables included in multivariate logistic regression*

## Discussion

The current study identified a high prevalence (79.3%) of drug-related problems among patients with kidney dysfunction. This is comparable to that identified in studies conducted elsewhere: 89.2% in the United Arab Emirates [28], 70.3% in Nigeria [12], 78.6%, and 82.3% in Ethiopia [24, 29].

However, the current prevalence is considerably lower than 100% in Kenya [23] and 100% in Indonesia [30]. This discrepancy in these two findings could be attributed to the fact the studies were conducted only in CKD patients while our study included both CKD and AKI patients. DRPs are more prevalent in CKD patients because of long-term therapy to manage disease progression and associated complications [11]. Additionally, the study in Indonesia had more than 80% of the participants in stage 5 CKD in which patients are likely to have more complications and, therefore, require more drugs to manage these complications, thereby increasing the possibility of DRPs.

On the other hand, the current prevalence is much higher than that reported in other studies 41.63% in France [31], 51.35%, and 29.41% in India [32, 33]. This could be partly explained by the different study designs used and the characteristics of the participants in those studies. Savitha et al. (2020) and Mongaret et al. (2020) conducted interventional studies thereby helping to prevent the number of new DRPs as the study proceeded. The study by Joel et al. (2013) recruited only patients on hemodialysis while the current study recruited all patients with kidney dysfunction including those not on dialysis. Management of patients on hemodialysis usually involves strict adherence to treatment guidelines and this could explain the low prevalence of DRPs in their study [34].

‘Untreated symptoms or indication’ was the most common DRP (35.6%) which is similar to proportions reported in studies in Ethiopia (31%) and France (30%) [35]. However, ‘untreated symptoms or indication’ accounted for much lower proportions in other studies: 8.3% in Ethiopia [28], 8.6% and 13.6% in India [29, 36]. This difference can be explained by the different healthcare practices with some of the studies conducted in settings with better access to diagnostic equipment, medication, and stringent guidelines for the management of kidney dysfunction. This enables early detection and therefore treatment of symptoms or indications. The other possible explanation for the higher proportion of ‘untreated symptoms or indication’ in this study is its prospective nature where active follow-up of patients can reveal more untreated symptoms, unlike the cross-sectional study by Legesse et al. (2022). The most common untreated conditions were diseases of the blood or blood-forming organs (28.1%), followed by clinical findings of the genitourinary system (17.2%). Untreated conditions usually arise due to the failure of physicians to focus on minor patient disease conditions, such as low hemoglobin, headache, constipation, diarrhea, electrolyte abnormalities, and pain while treating other major conditions [36]. If left untreated, diseases of the blood or blood-forming organs like anemia can lead to an increased risk of cardiovascular disease, rapid progression of the disease, reduced quality of life, and increased hospitalizations in patients with kidney dysfunction [37].

‘Adverse drug event (possibly) occurring’, accounting for 28.3% of all DRPs, was the second most commonly encountered. These findings are consistent with those of a study in India, 19% of all DRPs were ADRs in patients with renal compromise [38]. Kidney dysfunction has previously been identified as a predictor of ADRs [39]. Patients with kidney dysfunction often take multiple medications, of which polypharmacy has also been attributed to the occurrence of ADEs [40]. However, this proportion is much higher compared to 7.07% in Ethiopia [29]. This could be explained by the tools used for data collection in this study. The PCNE classification tool is designed to also capture possible adverse drug events thereby increasing the proportion of ‘adverse drug event (possibly) occurring’. ‘Inappropriate drug according to the guidelines was the major cause of ‘adverse event (possibly) occurring’. Certain drugs are generally contraindicated in patients with kidney dysfunction while others are not recommended below certain eGFR levels because of their potential for rapid disease progress and their accumulation in the body leading to toxicity [10]. Additionally, ‘dosage regimen too frequent’ and ‘drug dose too high’ were also major causes of ‘adverse event (possibly) occurring’. This can be attributed to the failure to adjust doses for renally excreted drugs according to the eGFR levels of the patients. This presents an opportunity for health workers to pay more emphasis on drug doses during prescription in the management of patients with kidney dysfunction.

‘Effect of drug treatment not optimal’ accounted for approximately 23.3% of the identified drug-related problems. Suboptimal treatment may result in reduced effectiveness and thereby poor therapeutic outcomes, increased treatment costs, and disease resistance to drug treatment [41]. This particularly concerning given that antimicrobials were the most common drugs (31.3%) involved in suboptimal treatment as this may contribute to antimicrobial resistance. With the major causes of the ‘effect of drug treatment not optimal’ being incomplete drug treatment (27.8%) and low drug doses (24.1%), this presents an opportunity for healthcare workers involved in prescribing to pay more attention to treatment guidelines and appropriate medication doses.

‘Unnecessary drug treatment’ accounted for 10.2% of the total DRPs. This finding is in line with previous studies: 14.2% in Ethiopia [29], 12.8% in France [35], and 18% in Kenya [23]. Some of the causes of ‘unnecessary drug treatment’ include a lack of a clear indication for the drug, duplication of therapeutic groups, and medication reconciliation problems, among others. Antimicrobials were the most common drugs given unnecessarily (32.1%) and this could lead to the development of antibiotic resistance, increased healthcare costs, and potential adverse events to the patients.

The identification of risk factors for drug-related problems in patients with kidney dysfunction helps in the early detection and management of DRPs in high-risk individuals. This study showed that patients who stayed in the hospital for five or more days had 6.39 times higher odds of experiencing a DRP compared to those who stayed for two or fewer days. This is consistent with the findings of previous studies [42, 43]. This can be explained by the fact that longer hospital stay increases patients’ exposure to drugs, changes in regimen, medication errors, and adverse drug reactions [44]. Additionally, prolonged hospitalization may lead to physiological and health status changes in patients, such as bed sores and hospital-acquired infections, requiring additional medication to treat these conditions, thereby increasing the risk of DRP occurrence. Therefore, effective medication management and regular monitoring of patients during their hospital stay can help reduce the incidence of DRPs.

Despite the strengths, this study has some limitations. The study employed a consecutive sampling technique which involved selecting participants as they became available and this may limit the generalizability of the study findings.

## Conclusion

The results of this study show a high burden of DRPs among patients with kidney dysfunction. Untreated indications and adverse events are the most frequent drug-related problems in this patient population. Additionally, antimicrobials are the most involved drugs in suboptimal drug treatment and unnecessary drug treatment posing a serious health challenge as a potential driver for antimicrobial resistance. Staying in the hospital for five or more days significantly increases the risk of drug-related problems among patients with kidney dysfunction. The high prevalence of DRPs in patients with kidney dysfunction and the potential impact on antimicrobial resistance underscores the importance of regular medication reviews and close monitoring of patients with renal dysfunction. Patients with renal dysfunction who stay longer in the hospital need to be closely monitored for prompt identification and management of DRPs.

## Data Availability

The data set and data collection tools used in this study are available upon reasonable request from the corresponding author.

## Abbreviations

ADE: Adverse Drug Event
ADR: Adverse Drug Reaction
AKI: Acute Kidney Injury
AOR: Adjusted Odds Ratio
ATC: Anatomical therapeutic chemical classification
CI: Confidence interval
CKD: Chronic Kidney Disease
COR: Crude Odds Ratio
DRP: Drug-Related Problem
eGFR: estimated Glomerular Filtration Rate
ESKD: End-Stage Kidney Disease
HIV: Human Immunodeficiency Virus
ICD: International Statistical Classification of Diseases
IQR: Interquartile range
KDIGO: Kidney Disease Improving Global Outcomes
MRRH: Mbarara Regional Referral Hospital
MUST: Mbarara University of Science and Technology
PCNE: Pharmaceutical Care Network Europe
REC: Research Ethics Committee
SD: Standard deviation
SPSS: Statistical Package for Social Sciences

## References

[1] Levey AS, Levin A, Kellum JA (2013). Definition and classification of kidney Diseases. Am J Kidney Dis.

[2] Levey AS, Atkins R, Coresh J, Cohen EP, Collins AJ, Eckardt KU et al. Chronic kidney disease as a global public health problem: Approaches and initiatives - A position statement from Kidney Disease Improving Global Outcomes. Kidney Int [Internet]. 2007;72(3):247–59. 10.1038/sj.ki.5002343. 10.1038/sj.ki.5002343 17568785

[3] Haileamlak A (2018). Chronic Kidney Disease is on the rise. Ethiop J Health Sci.

[4] World Health Organization. WHO - The top 10 causes of death [Internet]. 24 Maggio. 2020 [cited 2023 Mar 13]. p. 1–7. Available from: https://www.who.int/news-room/fact-sheets/detail/the-top-10-causes-of-death.

[5] Matsha TE, Erasmus RT. Chronic kidney disease in sub-Saharan Africa. Lancet Glob Heal [Internet]. 2019;7(12):e1587–8. 10.1016/S2214-109X(19)30467-X. 10.1016/S2214-109X(19)30467-X 31708128

[6] Kaze AD, Ilori T, Jaar BG, Echouffo-Tcheugui JB. Burden of chronic kidney disease on the African continent: A systematic review and meta-analysis [Internet]. BMC Nephrology. BioMed Central Ltd.; 2018. 2022;19:1–11. Available from: https://bmcnephrol.biomedcentral.com/articles/10.1186/s12882-018-0930-5. 10.1186/s12882-018-0930-5 PMC5984759 29859046

[7] Lunyera J, Stanifer JW, Ingabire P, Etolu W, Bagasha P, Egger JR (2016). Prevalence and correlates of proteinuria in Kampala, Uganda: a cross-sectional pilot study. BMC Res Notes.

[8] Hill NR, Fatoba ST, Oke JL, Hirst JA, O’Callaghan CA, Lasserson DS et al. Global prevalence of chronic kidney disease - A systematic review and meta-analysis [Internet]. PLoS ONE. Public Library of Science; 2016. 2021;11. p. e0158765. Available from: https://journals.plos.org/plosone/article?id=10.1371/journal.pone.0158765. 10.1371/journal.pone.0158765 PMC4934905 27383068

[9] Kerr M, Bedford M, Matthews B, O’donoghue D (2014). The economic impact of acute kidney injury in England. Nephrol Dial Transplant.

[10] Pichai E, Lakshmanan M. Drug elimination. In: Introduction to Basics of Pharmacology and Toxicology: Volume 1: General and Molecular Pharmacology: Principles of Drug Action [Internet]. StatPearls Publishing; 2019 [cited 2022 Feb 18]. p. 117–29. Available from: https://www.ncbi.nlm.nih.gov/books/NBK547662/.

[11] Cardone KE, Bacchus S, Assimon MM, Pai AB, Manley HJ. Medication-related Problems in CKD. Adv Chronic Kidney Dis [Internet]. 2010 Sep 1 [cited 2022 May 19];17(5):404–12. 10.1053/j.ackd.2010.06.004. 10.1053/j.ackd.2010.06.004 20727510

[12] Adibe MO, Igboeli NU, Ukwe CV (2017). Evaluation of drug therapy problems among renal patients receiving care in some tertiary hospitals in Nigeria. Trop J Pharm Res.

[13] Quintana-Bárcena P, Lord A, Lizotte A, Berbiche D, Lalonde L (2018). Prevalence and management of drug-related problems in chronic Kidney Disease patients by severity level: a subanalysis of a cluster randomized controlled trial in community pharmacies. J Manag Care Spec Pharm.

[14] PCNE V9.1. Classification for Drug related problems V9.1. Pharm Care Netw Eur Assoc [Internet]. 2020;V 9.1:1–10. Available from: https://www.pcne.org/upload/files/334_PCNE_classification_V9-0.pdf.

[15] Adem F, Abdela J, Edessa D, Hagos B, Nigussie A, Mohammed MA. Drug-related problems and associated factors in Ethiopia: a systematic review and meta-analysis. J Pharm Policy Pract [Internet]. 2021 Dec 1 [cited 2021 Nov 16];14(1):1–24. 10.1186/s40545-021-00312-z. 10.1186/s40545-021-00312-z PMC8077957 33902729

[16] Alruqayb WS, Price MJ, Paudyal V, Cox AR. Drug-Related Problems in Hospitalised Patients with Chronic Kidney Disease: A Systematic Review [Internet]. Drug Safety. Springer; 2021 [cited 2021 Nov 29];44:1041–58. Available from: https://link.springer.com/article/10.1007/s40264-021-01099-3. 10.1007/s40264-021-01099-3 34510389

[17] Westberg SM, Yarbrough A, Weinhandl ED, Adam TJ, Brummel AR, Reidt SL (2018). Drug Therapy Problem Severity Following Hospitalization and Association with 30-Day clinical outcomes. Ann Pharmacother.

[18] Roy DA, Shanfar I, Shenoy P, Chand S, Up N, Kc BR (2020). Drug-related problems among chronic Kidney Disease patients: a clinical pharmacist led study. Int J Pharm Res.

[19] Susilawati NM, Halimah E, Saidah S (2021). Pharmacists’ strategies to detect, resolve, and prevent DRPs in CKD patients. Pharmacia.

[20] Salgado TM, Moles R, Benrimoj SI, Fernandez-Llimos F (2012). Pharmacists’ interventions in the management of patients with chronic Kidney Disease: a systematic review. Nephrol Dial Transplant.

[21] Manley HJ, Cannella CA, Bailie GR, St. Peter WL. Medication-related problems in ambulatory hemodialysis patients: A pooled analysis. Am J Kidney Dis [Internet]. 2005 Oct [cited 2021 Nov 19];46(4):669–80. Available from: https://pubmed.ncbi.nlm.nih.gov/16183422/. 10.1053/j.ajkd.2005.07.001 16183422

[22] Haseeb A, Winit-Watjana W, Bakhsh ARR, Elrggal ME, Hadi MA, Mously AA et al. Effectiveness of a pharmacist-led educational intervention to reduce the use of high-risk abbreviations in an acute care setting in Saudi Arabia: A quasi-experimental study. BMJ Open [Internet]. 2016 Jun 1 [cited 2023 Apr 26];6(6). Available from: https://pubmed.ncbi.nlm.nih.gov/27311911/. 10.1136/bmjopen-2016-011401 PMC4916617 27311911

[23] Njeri LW, Ogallo WO, Nyamu DG, Opanga SA, Birichi AR. Medication-related problems among adult chronic kidney disease patients in a sub-Saharan tertiary hospital. Int J Clin Pharm [Internet]. 2018 May 1 [cited 2021 Nov 15];40(5):1217–24. Available from: https://link.springer.com/article/10.1007/s11096-018-0651-7. 10.1007/s11096-018-0651-7 29766391

[24] Garedow AW, Mulisa Bobasa E, Desalegn Wolide A, Kerga Dibaba F, Gashe Fufa F, Idilu Tufa B et al. Drug-Related Problems and Associated Factors among Patients Admitted with Chronic Kidney Disease at Jimma University Medical Center, Jimma Zone, Jimma, Southwest Ethiopia: A Hospital-Based Prospective Observational Study. Int J Nephrol. 2019;2019. 10.1155/2019/1504371 PMC6854244 31772774

[25] National Kidney Foundation. CKD-EPI Creatinine Eq, National Kidney Foundation. (2021) | National Kidney Foundation [Internet]. 2021 [cited 2023 Nov 27];241:2021–3. Available from: https://www.kidney.org/content/ckd-epi-creatinine-equation-2021.

[26] Sin‐Ho J. Stratified fisher’s exact test and its sample size calculation. Biom J. 2014;56(1):129–140. 10.1002/bimj.201300048. 10.1002/bimj.201300048 PMC3884832 24395208

[27] KDIGO. Guidelines | KDIGO [Internet]. 2018 [cited 2022 Feb 9]. Available from: https://kdigo.org/guidelines/.

[28] Shouqair TM, Rabbani SA, Sridhar SB, Kurian MT. Evaluation of Drug-Related Problems in Chronic Kidney Disease Patients. Cureus [Internet]. 2022 Apr 11 [cited 2023 Mar 15];14(4). Available from: http://www.pmc/articles/PMC9091809/. 10.7759/cureus.24019 PMC9091809 35573572

[29] Legesse ES, Muhammed OS, Hamza L, Nasir BB, Nedi T. Medication related problems among ambulatory patients with chronic kidney disease at St. Paul’s Hospital Millennium Medical College, Addis Ababa, Ethiopia. PLoS One [Internet]. 2022 Dec 1 [cited 2023 Mar 16];17(12 December). Available from: https://pubmed.ncbi.nlm.nih.gov/36455046/. 10.1371/journal.pone.0278563 PMC9714937 36455046

[30] Ramadaniati HU, Anggriani Y, Wowor VM, Rianti A (2016). Drug-related problems in chronic kidneys Disease patients in an Indonesian hospital: do the problems really matter?. Int J Pharm Pharm Sci.

[31] Mongaret C, Aubert L, Lestrille A, Albaut V, Kreit P, Herlem E et al. The Role of Community Pharmacists in the Detection of Clinically Relevant Drug-Related Problems in Chronic Kidney Disease Patients. Pharmacy [Internet]. 2020 May 22 [cited 2023 Mar 16];8(2):89. Available from: http://www.pmc/articles/PMC7355920/. 10.3390/pharmacy8020089 PMC7355920 32456115

[32] Joel JJ, Shastry MMM. CS. A Study on Drug Related Problems and Pharmacist Intervention in Patients Undergoing Haemodialysis in a Tertiary Care Hospital. Int Res J Pharm Appl Sci (IRJPAS) [Internet]. 2013 Oct 31 [cited 2023 Mar 16];3(5):263–5. Available from: https://www.scienztech.org/index.php/irjpas/article/view/561.

[33] Savitha RS, Ramesh M, Shetty MS, Kiran KK (2020). Drug-related problems and pharmacist interventions in inpatients with chronic Kidney Disease. Int J Res Pharm Sci.

[34] Kim H, Jeong IS, Cho MK. Effect of Treatment Adherence Improvement Program in Hemodialysis patients: a systematic review and Meta-analysis. Int J Environ Res Public Health. 2022;19(18). 10.3390/ijerph191811657 PMC9517018 36141929

[35] Belaiche S, Romanet T, Allenet B, Calop J, Zaoui P. Identification of drug-related problems in ambulatory chronic kidney disease patients: a 6-month prospective study. J Nephrol [Internet]. 2012 Sep [cited 2022 Feb 4];25(5):782–8. Available from: https://pubmed.ncbi.nlm.nih.gov/22322820/. 10.5301/jn.5000063 22322820

[36] Greeshma M, Lincy S, Maheswari E, Tharanath S, Viswam S (2018). Identification of drug related problems by clinical pharmacist in prescriptions with polypharmacy: a prospective interventional study. J Young Pharm.

[37] Mathias SD, Blum SI, Sikirica V, Johansen KL, Colwell HH, Okoro T. Symptoms and impacts in anemia of chronic Kidney Disease. J Patient-Reported Outcomes. 2020;4(1). 10.1186/s41687-020-00215-8 PMC7391458 32728779

[38] Castelino RL, Sathvik BS, Parthasarathi G, Gurudev KC, Shetty MS, Narahari MG. Prevalence of medication-related problems among patients with renal compromise in an Indian hospital. J Clin Pharm Ther [Internet]. 2011 Aug 1 [cited 2023 Mar 18];36(4):481–7. Available from: https://onlinelibrary.wiley.com/doi/full/10.1111/j.1365-2710.2011.01266.x. 10.1111/j.1365-2710.2011.01266.x 21535060

[39] Yadesa TM, Kitutu FE, Tamukong R, Alele PE. Predictors of hospital-acquired adverse drug reactions: a cohort of Ugandan older adults. BMC Geriatr [Internet]. 2022 Dec 1 [cited 2023 Mar 18];22(1):1–11. Available from: https://bmcgeriatr.biomedcentral.com/articles/10.1186/s12877-022-03003-9. 10.1186/s12877-022-03003-9 PMC9033930 35461224

[40] Shegena EA, Nigussie KA, Tamukong R, Lumori BAE, Yadesa TM. Prevalence and factors associated with adverse drug reactions among heart failure patients hospitalized at Mbarara Regional Referral Hospital, Uganda. BMC Cardiovasc Disord [Internet]. 2022 Dec 1 [cited 2023 Mar 18];22(1). Available from: https://pubmed.ncbi.nlm.nih.gov/36368954/. 10.1186/s12872-022-02937-7 PMC9650824 36368954

[41] Belfrage B, Koldestam A, Sjöberg C, Wallerstedt SM (2014). Prevalence of suboptimal drug treatment in patients with and without multidose drug dispensing - a cross-sectional study. Eur J Clin Pharmacol.

[42] Prajapati A, Ganguly B. Appropriateness of drug dose and frequency in patients with renal dysfunction in a tertiary care hospital: A cross-sectional study. J Pharm Bioallied Sci [Internet]. 2013 [cited 2021 Dec 16];5(2):136–40. Available from: https://pubmed.ncbi.nlm.nih.gov/23833519/. 10.4103/0975-7406.111829 PMC3697192 23833519

[43] Saleem A, Masood I, Khan TM. Clinical relevancy and determinants of potential drug&ndash;drug interactions in chronic kidney disease patients: results from a retrospective analysis. Integr Pharm Res Pract [Internet]. 2017 Feb [cited 2021 Dec 16];6:71–7. Available from: https://www.pmc/articles/PMC5774325/10.2147/IPRP.S128816 PMC5774325 29354553

[44] Hohl CM, Kuramoto L, Yu E, Rogula B, Stausberg J, Sobolev B. Evaluating adverse drug event reporting in administrative data from emergency departments: A validation study. BMC Health Serv Res [Internet]. 2013 Nov 12 [cited 2023 Mar 21];13(1):1–11. Available from: https://bmchealthservres.biomedcentral.com/articles/10.1186/1472-6963-13-473. 10.1186/1472-6963-13-473 PMC3842633 24219303

